# The segmentation of the Argentine healthcare system in the care of patients with epidermolysis bullosa: challenges and proposals for a comprehensive model

**DOI:** 10.1007/s12687-025-00840-0

**Published:** 2025-11-17

**Authors:** Juan Manuel Martínez-Ripoll, Yolanda de la Fuente Robles, Marta García-Domingo

**Affiliations:** 1 DEBRA Spain, Social Worker, Jaén, España; 2 University of Jaén, Jaén, España

**Keywords:** Argentina, Challenges, Epidermolysis bullosa, Interdisciplinary care, Healthcare system

## Abstract

Epidermolysis bullosa is a rare genetic disorder characterized by extreme mucocutaneous fragility. The healthcare coverage in Argentina is divided into the public sector, social security, and the private sector. This study examines how this segmentation affects the socio-healthcare management of patients with EB, identifying barriers and challenges. A descriptive phenomenological qualitative design was employed. The sample included 91 participants: parents of minors with EB (*n* = 54), adults with EB (*n* = 26), and socio-healthcare professionals (*n* = 11). Data were collected through semi-structured interviews and open-ended online surveys. A reflexive thematic analysis was conducted using ATLAS.ti software. Health system segmentation negatively impacts the care of individuals with EB, alongside other factors such as deficient interdisciplinary coordination, the lack of protocols for transitioning from pediatric to adult care, centralization and scarcity of specialized services, and significant disparities in access to wound-care products and treatments. Healthcare inequities exacerbate the vulnerability of individuals with EB and their families. The disparities in socio-healthcare access for individuals with EB in Argentina are closely linked to the segmentation of the healthcare system. While the National Program for Rare Diseases represents progress, there remains an urgent need to implement a national plan that ensures equitable access to treatment, interdisciplinary teams, and specialized training.

## Introduction

### Epidermolysis bullosa: types, epidemiology, and complications

Epidermolysis Bullosa (hereafter, EB) is a heterogeneous group of rare genodermatoses characterized by extreme mucocutaneous fragility, leading to blistering and/or erosions even with minimal trauma (Bardhan et al. 2020). To date, more than 30 EB subtypes have been identified, varying in severity and classified based on the extent of blistering and/or erosions (Maseda Pedrero et al. 2021). As a result, diagnosis is complex, and classification has undergone multiple revisions (Has et al. 2021). According to the most recent consensus classification, EB is categorized into four major types: EB Simplex (EBS), Dystrophic EB (DEB), Junctional EB (JEB) and Kindler EB (KEB) (Has et al. 2021). Recent epidemiological studies in Argentina have reported the following distribution of prevalent cases: 708 living patients (328 EBS, 316 DEB, 43 indeterminate EB, 13 JEB and 8 KEB) (Valinotto et al. 2025).

Clinical manifestations range from localized blistering and/or erosions on the hands and feet to severe extracutaneous complications (Maseda et al. 2020), with squamous cell carcinoma being the leading cause of mortality. These physical manifestations are also accompanied by psychosocial and economic (Fine and Hintner 2009) factors that significantly impact the quality of life of individuals with EB (Salamon et al. 2025).

Due to the chronic and incurable nature of EB, current management strategies prioritize a multidisciplinary palliative care approach aimed at optimizing symptomatic management and addressing the psychosocial dimensions of the patient and their environment (Martínez-Ripoll et al. 2024; Popenhagen et al. 2023; Van Scheppingen et al. 2008). This approach underscores the importance of coordination among professionals from various disciplines, including dermatology, pediatrics, nursing, psychology and social work (Reimer et al. 2018), who play a fundamental role in the comprehensive management of the disease.

### The Argentine healthcare system and its care for individuals with epidermolysis Bullosa

The Argentine healthcare system is organized into three main sectors: the public sector, social security and the private sector (Belló and Becerril-Montekio 2011).

- The public sector provides free healthcare services to all individuals, with a particular focus on those without Social Security coverage or the financial means to afford private care (Arce 2012). However, it faces significant challenges, including resource shortages, limited infrastructure, and a strong dependence on provincial budgets (Belló and Becerril-Montekio 2011; Cavagnero and Bilger 2010).
- The social security sector, which covers formal workers and their families through mandatory contributions from both employees and employers (Arce 2012). Despite its extensive coverage, there are significant disparities in access and quality of care among different social health insurances, largely influenced by the type of employment (Nievas et al. 2021). Some social health insurances redirect higher-income affiliates to private health insurance companies, further contributing to the segmentation of the system (Acuña and Chudnovsky 2002; Cavagnero and Bilger 2010).
- The private sector includes clinics, hospitals, and private health insurance companies, which provide services to individuals who can afford private care (Arce 2012). This sector is also characterized by significant inequalities, as the high costs of private health insurance companies limit access for a large portion of the population (Acuña and Chudnovsky 2002; Cavagnero and Bilger 2010).

This segmentation and inequality within the healthcare system have an even greater impact on the management of rare diseases such as EB, given the complex and specific needs these conditions entail (Nievas et al. 2021).

To address these challenges, Law 26.689 was enacted in 2011 and regulated in 2015, aiming to ensure comprehensive care for individuals with rare diseases through the National Program for Rare Diseases (FADEPOF 2018).

## Current study

The primary objective of this research was to analyze the healthcare and social care provided to individuals with EB within the Argentine healthcare system, specifically investigating potential inequalities stemming from systemic segmentation. More precisely, the study explored disparities in access to and quality of healthcare services based on the type of health coverage.

This research was guided by the central question: How does the segmentation of the Argentine healthcare system influence access to and quality of healthcare services for individuals with Epidermolysis Bullosa?

## Methods

### Study design

This study employed a qualitative, descriptive phenomenological research design, focusing on the lived experiences of parents, individuals with EB, and healthcare professionals regarding the care provided by the Argentine healthcare system, as well as their perceptions, emotions, and the meanings they attribute to these experiences (Wojnar and Swanson 2007). All procedures followed were in accordance with the ethical standards of the responsible committee on human experimentation (institutional and national) and with the Helsinki Declaration of 1975, as revised in 2000. Informed consent was obtained from all patients for being included in the study.

### Participants, procedure and instruments

The final sample consisted of 91 participants, distributed as follows: 54 parents (47 mothers and 7 fathers) of minors diagnosed with EB, 26 adults diagnosed with EB, and 11 healthcare and social care professionals (Table 1). Informed consent was obtained, ensuring confidentiality and anonymity.

**Table 1:** Participant characteristics (*N* = 91)

	*N* = 91	%
Parents and adults with EB	80	72.6
Mother of a child with EB	47	42.7
Father of a child with EB	7	6.3
Woman with EB	21	19.1
Man with EB	5	4.5
Health coverage		
Public sector	14	12.7
Social security	48	43.6
Private sector	18	16.3
Type of EB		
DEB	57	51.8
EBS	23	20.9
Employment status		
Employee	48	43.6
Unemployed	23	20.9
Pensioner	9	8.1
Province		
Buenos Aires	41	37.3
Entre Ríos	5	4.5
Mendoza	6	5.4
Catamarca	1	0.9
Córdoba	13	11.8
San Juan	4	3.6
Santa Fe	8	7.2
Chubut	1	0.9
Corrientes	1	0.9
Social and healthcare professionals	11	27.4
Man	0	0
Woman	11	100
Discipline		
Dermatology	7	6.3
Psychology	3	2.7
Plastic surgery	1	0.9
Area of care		
Private sector	2	1.8
Public sector	9	8.1
Province		
Buenos Aires	8	7.2
Salta	1	0.9
Chaco	1	0.9
Mendoza	1	0.9

Abbreviations: *EB* Epidermolysis bullosa, *DEB* Dystrophic Epidermolysis Bullosa, *EBS* Epidermolysis bullosa Simplex

To achieve a representative analysis, all EB subtypes from various provinces across Argentina were included, and professionals from both public and private healthcare sectors participated.

In September 2024, two semi-structured interviews were conducted to explore and gather information directly from key stakeholders in the Argentine context:

- The first interview was conducted with an adult diagnosed with Dystrophic EB (DEB), using an interview guide structured into five thematic areas: healthcare and social care services, physician-patient communication, psycho-emotional support, access to resources and challenges, and recommendations for improvement.
- The second interview was conducted with a dermatologist from the public sector of Buenos Aires (Argentina), using an interview guide structured into five thematic areas: clinical protocols and practice, training and knowledge, multidisciplinary approaches, psychosocial support, and barriers and improvements.

Based on the insights obtained from these interviews and applying the expert judgment technique, three online open-ended surveys were designed and adapted to the Argentine context, targeting the following participant groups:

1. Parents of minors diagnosed with any EB subtype residing in Argentina, regardless of their health coverage.
2. Adults diagnosed with any EB subtype residing in Argentina, regardless of their health coverage.
3. Healthcare and social care professionals working in both public and private sectors, who provide care for at least one child or adult diagnosed with EB in Argentina.

The involvement of experts in this process ensured that the surveys were tailored to the specific realities and needs of the participants.

Using purposive sampling, families and professionals were contacted, and additional participants were recruited through snowball sampling.

- The parent survey included 34 questions organized into seven thematic areas: healthcare and social care services, interprofessional coordination, treatment and resources, household impact, family planning, recommendations, and improvements. For adults with EB, an additional thematic area on employment was included.
- The healthcare and social care professionals’ survey included 16 questions across seven thematic areas: clinical protocols and practice, training and knowledge, multidisciplinary approaches, psychosocial support, barriers to care, professional impact, and recommendations for improvement.

The survey design was based on validated scales such as the Hospital Consumer Assessment of Providers and Systems (HCAHPS), as well as the information gathered from key informants through exploratory semi-structured interviews and previous research on the topic (Kearney et al. 2020; Togo et al. 2025; Reimer et al. 2018). The surveys were distributed via Google Forms between September and November 2024, and the data were exported using Microsoft Excel. Surveys were answered in Spanish and quotations were translated into English by the authors.

**Fig. 1:**
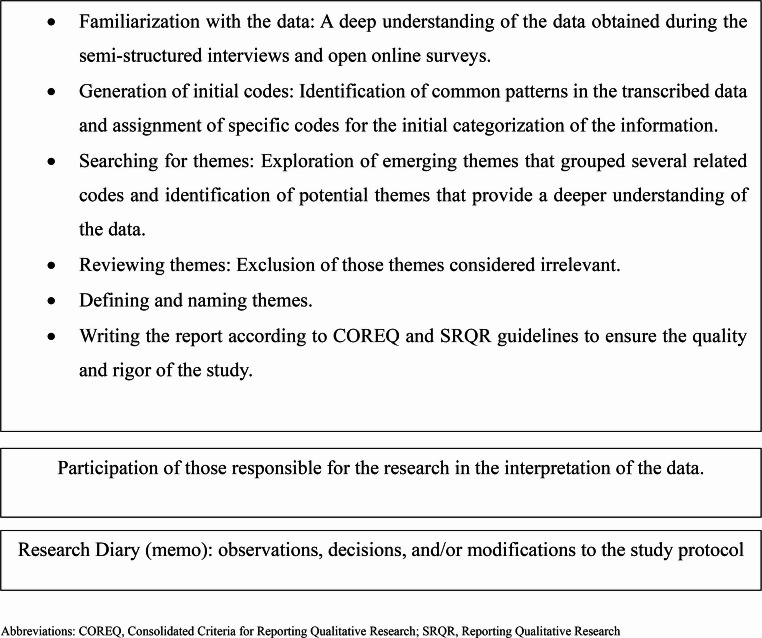
Data management and analysis. Abbreviations: COREQ, Consolidated Criteria for Reporting Qualitative Research; SRQR, Reporting Qualitative Research

The collected data were analyzed using a reflexive thematic approach (Braun and Clarke 2021) (Fig. 1).

First, JMM-R conducted an in-depth review of the information obtained to ensure accuracy. Common patterns were identified, and JMM-R applied the initial coding to classify the data, while MG-D and YDLFR reviewed and refined the coding to enhance reliability. To further strengthen the process, all authors (JMM-R, MG-D, and YDLFR) maintained a research diary (memo) to record personal reflections, including observations, decisions, and adjustments to the research design (Johnson et al. 2020; Tong et al. 2007). Finally, JMM-R organized the results into two main areas of analysis, with feedback and validation from MG-D and YDLFR:

- Social and healthcare intervention for Epidermolysis bullosa in Argentina.
- Inequality in access to healing and treatment products for patients with Epidermolysis bullosa in Argentina.

For transcription and content analysis, the qualitative data analysis software ATLAS.ti (version 25.0.1) was used, adhering to the guidelines established by Reporting Qualitative Research (SRQR) and the Consolidated Criteria for Reporting Qualitative Research (COREQ) (Dossett et al. 2021).

To ensure confidentiality, all information was anonymized and coded, as stipulated in the informed consent document signed by the research participants. The coding system was structured as follows:

- Parents were identified as Father (Father) or Mother (Mother), followed by the child’s age (Xa), EB type (categorized as EBS or EBD), and healthcare coverage classification: Public sector (Pub), Social security (SS), and Private sector (Priv).
- Adults with EB were coded as Man (Man) or Woman (Woman), followed by their EB type (EBS or EBD) and healthcare coverage classification (Pub, SS, Priv).
- Healthcare and social professionals were identified by their specialty: Dermatology (DERM), Plastic Surgery (PS), and Psychology (PSI), followed by their healthcare sector: Public (Pub) or Private (Priv).

Each element was separated by a forward slash (/) and followed by a number in parentheses, corresponding to the assigned participant number.

## Results

### Sociosanitary intervention in epidermolysis bullosa in Argentina

**Fig. 2:**
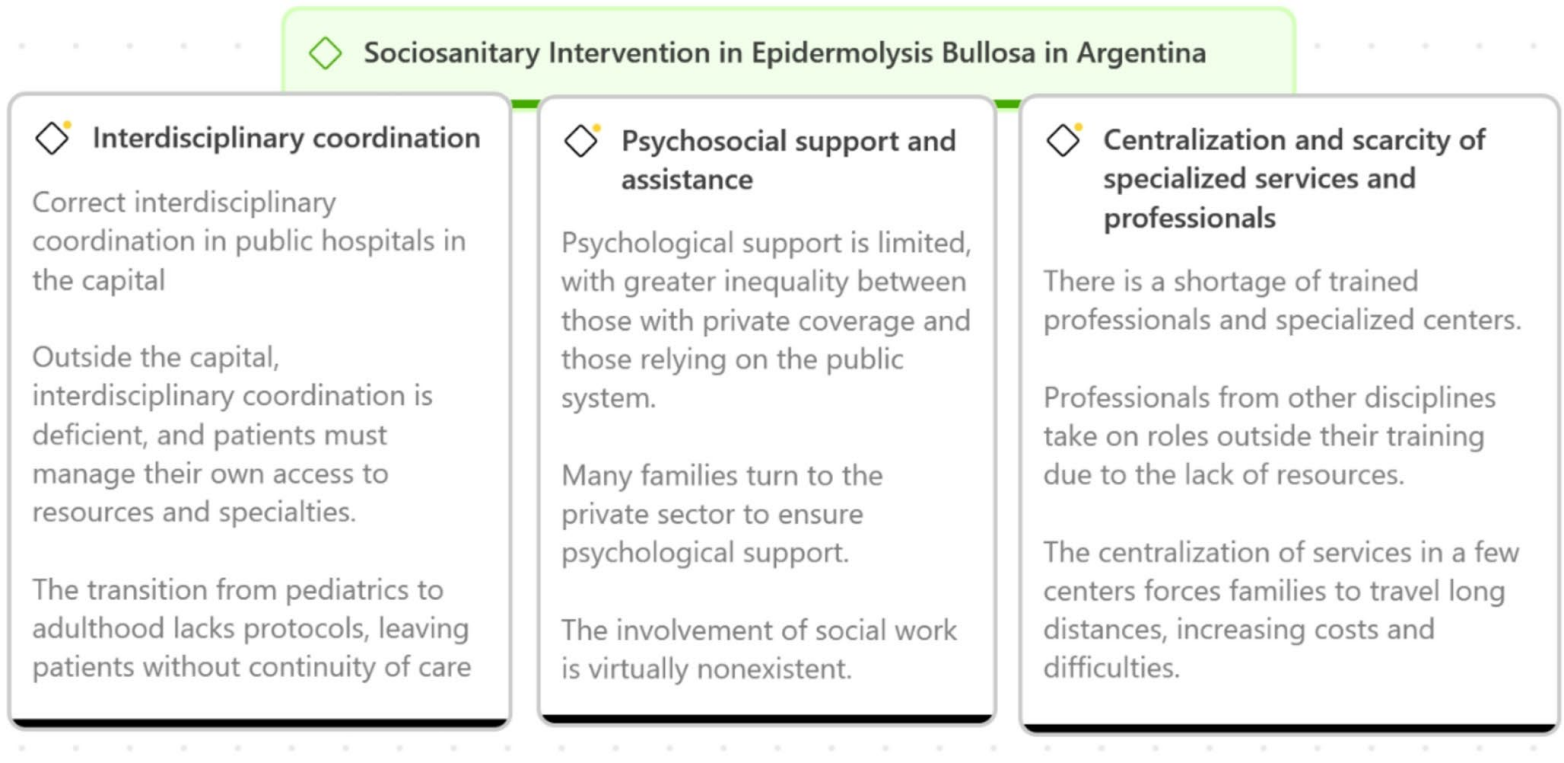
Social and healthcare intervention for Epidermolysis bullosa in Argentina

Healthcare services for patients with EB in Argentina exhibit significant deficiencies due to the segmentation and fragmentation of the healthcare system (Fig. 2).

## Interdisciplinary coordination

Public hospitals in Argentina’s capital have successfully implemented effective coordination models that integrate various specialties into joint sociosanitary decision-making, enabling a more structured approach aligned with the complex needs of individuals with EB and their families.

“At Hospital Garrahan, an interdisciplinary approach is adopted, involving doctors, nurses, therapists, social workers, and specialists to provide better patient care.” DERM/Pub (81).

”At Hospital de Clínicas José de San Martín, we use a patient history specifically developed for EB patients. A checklist is implemented to ensure that no aspect is overlooked during the examination. Based on the diagnosis, referrals and multidisciplinary evaluations are arranged.” DERM/Pub (82).

However, this reality is not uniform across all hospitals in the capital, nor in those located outside the province, where deficiencies in interdisciplinary coordination and resource access significantly limit the quality of care provided.

“Patients are usually responsible for handling paperwork and explaining their condition themselves. Although we offer our contact details for other sociosanitary professionals to reach out, this has never happened in five years.” PSI/Pub (86).

”In the hospital where my daughter was born, we were taught an incorrect bandaging technique, and the staff had minimal preparation to handle her condition. Additionally, there was little interest shown. After discharge, we continued care at Hospital Garrahan.” Mother/2a/EBS/SS (2).

This issue worsens during the transition from pediatric to adult care, a critical period characterized by the absence of formal protocols to ensure continuity of care. As a result, adult patients are left unprotected, assuming responsibility for managing access to resources and coordinating care across specialties themselves or delegating these tasks to their caregivers—a function that should be formally structured within the healthcare system.

“When I was a child, multidisciplinary care was available in public hospitals. As an adult, I coordinate my own care and act as the link between specialists.” Woman/EBD/Pub (61).

### Psychosocial support and assistance

Psychological support for pediatric patients is significantly limited and largely dependent on the type of healthcare coverage available. Families with social security coverage have better chances of accessing such support; however, they are often forced to resort to private services to ensure specialized care, highlighting the insufficiency of public resources allocated to this area.

“Overcoming challenges requires solid psychological support, acceptance of the disease, and working on all aspects that contribute to improving the patient’s quality of life.” DERM/Priv (83).

“For me, psychological support is just as important as medication and treatment. Individuals with EB must not only cope with their disease but also with the way others perceive them, which is often painful due to a lack of awareness.” Mother/6a/EBD/SS (5).

“My daughter has always had to see a psychologist privately; our health insurance has never covered it, nor has it ever been offered at the hospital.” Mother/12a/EBD/SS (15).

Similarly, social work intervention is notably restricted, focusing almost exclusively on the initial management of administrative procedures. This limited engagement lacks continuity and follow-up, leaving families without the comprehensive support needed to navigate the emotional, social, and economic challenges associated with caring for a child with EB.

“Social work intervention is a major issue. Similar to psychological services, our institution cannot meet patient demand.” DERM/Pub (84).

“I have always been denied social work assistance.” Father/4a/EBD/Pub (13).

Psychosocial support for adults is even more deficient than in the pediatric setting. The lack of clear referral points, specialized teams, and disparities in healthcare coverage exacerbate this situation. Individuals with access to private healthcare receive better services, while those relying solely on the public system face significant barriers to accessing and maintaining care. The analysis indicates that inequalities in access to psychosocial support are even more pronounced in adult patients than in pediatric cases.

“At this point, I cannot afford it. I have several mental health diagnoses, but accessing treatment is financially impossible, especially with the cost of medication. The resignation to inadequate healthcare is one of the most painful aspects of my country’s current reality.” Woman/EBD/Pub (65).

Access to psychological services is nearly restricted to the private sector, leaving a significant portion of the population without viable options. Furthermore, social work intervention is almost nonexistent, limited to exceptional cases that do not address the everyday needs of individuals with EB.

“I have never received any social work assistance or had it offered to me.” Man/EBD/SS (72).

### Centralization and scarcity of specialized services and professionals

The analysis highlights a shortage of resources and lack of specialty integration, forcing professionals from other disciplines to take on responsibilities beyond their training, underscoring the precariousness and fragmentation of the healthcare system.

“There is a lack of trained personnel and healthcare centers to provide appropriate monitoring and treatment. Additionally, only those with social security coverage or financial means have access to specialized products.” PS/Pub (90).

“I have never had a dedicated nurse throughout my life. In Argentina, at least in my experience, this role is not part of the care model. My dermatologist has always been my primary healthcare provider.” Woman/EBD/Pub (55).

The country’s vast geography further exacerbates the situation, making continuous patient follow-up difficult. Due to the scarcity of specialized services and the centralization of resources, many families are forced to travel to another hospital or even another province to receive adequate medical care. This necessity increases both the financial and emotional burden associated with treatment, adding another layer of difficulty for individuals with EB and their families.

“More healthcare training is needed across all specialties. There should be more specialized centers, not just one.” Mother/13a/EBD/SS (36).

“There is a lack of treatments, products, and specialized centers for interdisciplinary evaluations. Law 26.689 should be enforced.” DERM/Priv (85).

This reality not only impacts the quality of care but also highlights deep inequalities in the availability and access to fundamental resources.

### Inequality in access to blister healing and treatment products for patients with epidermolysis bullosa in Argentina

**Fig. 3:**
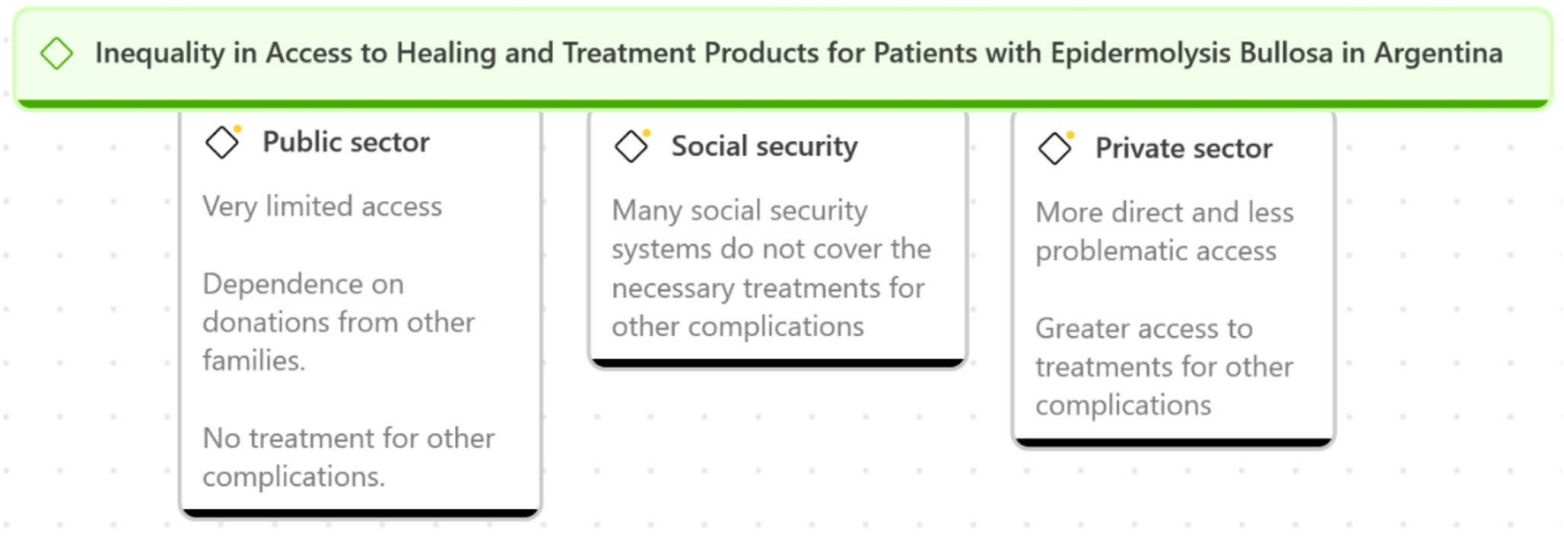
Inequality in access to healing and treatment products for patients with Epidermolysis bullosa in Argentina

Access to essential healing materials and treatment for managing complications in patients with EB reflects deep inequalities within the Argentine healthcare system, impacting both pediatric and adult populations (Fig. 3).

For pediatric patients, access to healing materials and other necessary products or treatments largely depends on the type of health coverage. In the public system, families frequently rely on donations from other families to meet these basic care needs. Those with social security coverage face bureaucratic processes to obtain them. In contrast, private sector users have more direct and less problematic access, highlighting the inequality across different health coverage systems.

“I have never been able to get my social security to cover the healing materials and my treatment; it’s a constant struggle. Even the social security employees recommend that I do it through a lawyer.” Mother/16 years/EB/SS (35).

“Other families send me donations since I don’t have social security or prepaid healthcare.” Mother/4 years/EB/Public (36).

“The main challenge for families and professionals is the high demand for healing products and the refusal of social security to cover both basic and new treatments.” DERM/Public (82).

In the adult population, access to essential healing products is not the only challenge. Employment integration becomes crucial, as formal employment may be the only route to access social security and, therefore, the necessary products and treatments. Adults with EB face significant barriers in obtaining and maintaining employment due to the physical limitations and continuous care needs associated with their condition.

“I am very scared for the future. I fear not having enough money to avoid dying from an infection or to afford my treatment. I pray every day that nothing with my disease gets worse because I can’t buy anything. I fear dying because I can’t pay for my survival.” Woman/EB/SS (79).

“When I have an interview and mention that I have a disease, my profile is rejected. I would love to work; it’s one of my big dreams.” Woman/EB/Public (70).

This shortage forces the families of both pediatric and adult patients to rely on local third-sector organizations or donations from other families to compensate for the deficiencies of the public system. However, these initiatives constitute temporary solutions that do not ensure stable or sustainable access over time.

“The only way to access treatment is through donations or redistribution among the patients themselves.” Woman/EB/Public (57).

“Third-sector entities are a very important pillar in providing support and mutual assistance.” DERM/Public (82).

In addition to essential healing products, treatment for managing other complications, particularly pain, is one of the primary concerns in both populations. The lack of specific protocols leaves families depending on home remedies, an inadequate solution that profoundly impacts the quality of life of patients and their caregivers.

“I am currently not receiving treatment from the hospital, and social security doesn’t cover anything, so sometimes I resort to home remedies like applying salt to the wounds.” Woman/EB/Public (62).

The lack of specific training in palliative care and the absence of clear national policies perpetuate a situation of extreme vulnerability, leaving many patients without relief from their daily suffering.

“We need a protection law that fits the reality of EB and covers all the needs for a dignified life.” Woman/EB/Public (66).

“The main challenge is the availability of healing products for patients and the need for related specialties. We should have a national coverage plan, as happens with other diseases.” DERM/Public (83).

The testimonies reveal distress, fear, and frustration in the face of a healthcare system that does not cover their basic needs, where both people with EB and professionals demand a national plan that guarantees comprehensive care and a dignified life for all individuals with EB.

## Discussion

This study makes a significant contribution to the understanding of the socio-healthcare provision for patients with EB in Argentina, providing a detailed and updated view of the reality experienced by the participants. The study involved 54 parents (47 mothers and 7 fathers) of minors diagnosed with EB, 26 adults diagnosed with EB, and 11 socio-healthcare professionals.

Through its qualitative approach, this study confirms that the segmentation of the Argentine healthcare system (Acuña and Chudnovsky 2002) significantly influences the quality of socio-healthcare provision (Cavagnero and Bilger 2010), generating inequalities between different health coverage systems (Arce 2012). Other factors that widen the care gap for individuals with EB and their families, such as the absence of national protocols and the scarcity of available resources (Belló and Becerril-Montekio 2011), along with the barriers patients face in accessing wound-care products and treatments (FADEPOF 2018), have also been insufficiently studied in Argentina and are addressed here to identify key areas for improving EB management.

It is imperative to implement effective coordination across levels of care through interdisciplinary teams trained in EB (Mauritz et al. 2021; Prodinger et al. 2019). Additionally, strengthening psychosocial support via public programs that assist both families and patients is essential to reduce reliance on private services (Cavagnero and Bilger 2010). The establishment of clear, standardized protocols, together with a national coverage plan that guarantees equitable access to treatments and wound-care products, is fundamental for addressing the needs of both pediatric and adult populations, the latter facing challenges that are exacerbated not only by system segmentation but also by the lack of age-specific care pathways and resources tailored to this stage of life.

In contrast, neighboring countries exhibit a more consolidated approach to public policies for EB. In Chile, the disease is explicitly included in the Ricarte Soto Law (Ley N.º 20.850, 2015), which ensures coverage for high-cost treatments and products. In Brazil, the Clinical Protocol and Therapeutic Guidelines (Togo et al. 2025) sets out multidisciplinary treatment standards, allows flexible use of dressings according to clinical need, and provides comprehensive patient follow-up. Furthermore, in both contexts, organizations such as DEBRA (an international patient association aimed at improving the quality of life of individuals with EB and their families) complement these policies by supporting patients and their families, guiding healthcare professionals, and advocating for patients’ rights.

In Argentina, the absence of specific public policies for EB, coupled with the lack of a national DEBRA organization, heightens the vulnerability of patients and their families. Participant testimonies reflect widespread distress and underscore the urgent need for public strategies that ensure equitable, high-quality access to treatments, essential products, and interdisciplinary palliative care, regardless of socioeconomic status or geographic location.

Future research could focus on optimizing the structure of comprehensive care teams and improving access to critical resources for all individuals with EB, independently of health coverage.

### Strengths and limitations

The study’s limitations include the sample size and diversity. Although 91 participants took part, the majority were woman, which could introduce a bias in the representation of family and personal experiences. Additionally, the use of snowball sampling may have restricted the scope of recruitment, potentially leaving out other participants. Lastly, the geographical context also poses a limitation, as regional disparities in infrastructure, quality of care, and professional training are not always fully reflected, and participation was significantly lower outside of the capital. Nevertheless, this study captures and analyses the experiences of 91 individuals living with or facing the reality of a specific rare disease in Argentina, a considerable sample size given the well-documented challenges in accessing this population. This not only underscores the robustness of the data but also highlights the study’s unique contribution to a largely under-researched area in the country.

### Implications

The National Rare Diseases Program represents progress; however, its implementation faces critical challenges, exacerbated by the absence of a consolidated national approach and the lack of standardized protocols.

In light of this, the findings from this study highlight the urgent need for a comprehensive approach to the care of individuals with EB and their families, particularly during the transition from pediatric to adult care. Public policies must ensure equitable access to treatments and essential products, especially for those with fewer resources, and address the inequalities generated by different health coverage systems. The implementation of a national plan that integrates interdisciplinary teams, standardized protocols, and improved professional training in key areas such as EB, palliative care, and interdisciplinary coordination is recommended.

## Conclusions

The aim of this study was to analyze the socio-healthcare provision for individuals with EB within the Argentine healthcare system, investigating the inequalities generated by the segmentation inherent to the system. Through qualitative analysis, it can be concluded that the segmentation of the Argentine healthcare system affects the access and quality of socio-healthcare for individuals with EB and their families. However, other structural factors, such as the scarcity of specialized centers, the limited availability of trained professionals, the lack of psychosocial support, and difficulties in accessing wound-care products, also contribute to the care gaps. Differences in health coverage and geographic inequalities limit access to essential treatments and resources, generating notable inequities. Despite the important role of existing programs and organizations, there is no specific entity in Argentina that intervenes in a specialized and comprehensive manner in the care of EB. While the National Rare Diseases Program represents progress, the need remains for an effective national plan that ensures comprehensive and equitable care, addressing both system segmentation and the other structural variables that influence the quality of care.

## Data Availability

The data that support the findings of this study are available on request from the corresponding author. The data are not publicly available due to privacy or ethical restrictions.
